# Late Cardiac Events after Childhood Cancer: Methodological Aspects of the Pan-European Study PanCareSurFup

**DOI:** 10.1371/journal.pone.0162778

**Published:** 2016-09-19

**Authors:** Elizabeth A. M. Feijen, Anna Font-Gonzalez, Elvira C. van Dalen, Helena J. H. van der Pal, Raoul C. Reulen, David L. Winter, Claudia E. Kuehni, Riccardo Haupt, Daniela Alessi, Julianne Byrne, Edit Bardi, Zsuzsanna Jakab, Desiree Grabow, Stanislaw Garwicz, Momcilo Jankovic, Gill A. Levitt, Roderick Skinner, Lorna Zadravec Zaletel, Lars Hjorth, Wim J. E. Tissing, Florent de Vathaire, Mike M. Hawkins, Leontien C. M. Kremer, PanCareSurFup consortium

**Affiliations:** 1 Department of Pediatric Oncology, Emma Children’s Hospital/ Academic Medical Center Amsterdam, meibergdreef 9, 1105 AZ, Amsterdam, the Netherlands; 2 Department of Medical Oncology, Academic Medical Center Amsterdam, meibergdreef 9, 1105 AZ, Amsterdam, the Netherlands; 3 Department of Public Health, Epidemiology and Biostatistics, Centre for Childhood Cancer Survivor Studies, School of Health and Population Sciences, Public Health Building, University of Birmingham, Birmingham, B15 2TT, United Kingdom; 4 Swiss Childhood Cancer Registry, Institute of Social and Preventive Medicine, University of Bern, Finkenhubelweg 11, 3012, Bern, Switzerland; 5 Epidemiology and Biostatistics Section, Gaslini Children Hospital, Via Gerolamo Gaslini, 5, 16148, Genova, Italy; 6 Childhood Cancer Registry of Piedmont, Cancer Epidemiology Unit, Citta' della Salute e della Scienza Hospital-University of Turin and Center for Cancer Prevention (CPO), Via Santena 7, 10126, Torino, Italy; 7 Boyne Research Institute, Tiernan House, Fair Green, Drogheda, Ireland; 8 2nd Department of Pediatrics, Semmelweis University, Üllői út 26, 1085, Budapest, Hungary; 9 Department of Pediatric Oncology, Markusovszky Hospital, Markusovszky Lajos u. 5, 9700, Szombathely, Hungary; 10 German Childhood Cancer Registry (GCCR), Institute of Medical Biostatistics, Epidemiology and Informatics, University Medical Center, Mainz, Germany; 11 Department of Pediatrics, Skåne University Lund, Getingevägen 4, 222 41, Lund, Sweden; 12 Pediatric Hematology Unit, San Gerardo Hospital, Via Primo Maggio, 22, 38089, Monza, Italy; 13 Department of Paediatric and Adolescent Haematology and Oncology, and Children's BMT Unit, Great North Children's Hospital, Newcastle, United Kingdom; 14 Department of Paediatric and Adolescent Haematology and Oncology, and Children's BMT Unit, Great North Children's Hospital, and Northern Institute of Cancer Research, Newcastle University, Newcastle, United Kingdom; 15 Division of Radiotherapy, Institute of Oncology, Zaloška cesta 2 SI– 1000, Ljubljana, Slovenia; 16 Department of Pediatric Oncology, University of Groningen, University Medical Center Groningen, Groningen, the Netherlands; 17 Radiation Epidemiology Group, Gustave Roussy, Inserm, UMR1018, Villejuif, France; Universita degli Studi di Parma, ITALY

## Abstract

**Background and Aim:**

Childhood cancer survivors are at high risk of long-term adverse effects of cancer and its treatment, including cardiac events. The pan-European PanCareSurFup study determined the incidence and risk factors for cardiac events among childhood cancer survivors. The aim of this article is to describe the methodology of the cardiac cohort and nested case-control study within PanCareSurFup.

**Methods:**

Eight data providers in Europe participating in PanCareSurFup identified and validated symptomatic cardiac events in their cohorts of childhood cancer survivors. Data on symptomatic heart failure, ischemia, pericarditis, valvular disease and arrhythmia were collected and graded according to the Criteria for Adverse Events. Detailed treatment data, data on potential confounders, lifestyle related risk factors and general health problems were collected.

**Results:**

The PanCareSurFup cardiac cohort consisted of 59,915 5-year childhood cancer survivors with malignancies diagnosed between 1940 and 2009 and classified according to the International Classification of Childhood Cancer 3. Different strategies were used to identify cardiac events such as record linkage to population/ hospital or regional based databases, and patient- and general practitioner-based questionnaires.

**Conclusion:**

The cardiac study of the European collaborative research project PanCareSurFup will provide the largest cohort of 5-year childhood cancer survivors with systematically ascertained and validated data on symptomatic cardiac events. The result of this study can provide information to minimize the burden of cardiac events in childhood cancer survivors by tailoring the follow-up of childhood cancer survivors at high risk of cardiac adverse events, transferring this knowledge into evidence-based clinical practice guidelines and providing a platform for future research studies in childhood cancer patients.

## Introduction

Treatment for children with cancer has improved considerably over the last decades, resulting in better survival.[1] This success has brought to light the wide variety of late complications and the long-term adverse effects of cancer and its treatment. Approximately 75% of childhood cancer survivors (CCS) develop at least one chronic health condition such as long-term cardiac, endocrine, neurologic or psychosocial effects.[2–4]

Symptomatic cardiac events (CEs) such as heart failure, cardiac ischemia, arrhythmia, pericarditis and valvular disease are well-known long-term side effects of treatment for childhood cancer, which can lead to long-term morbidity and early mortality among CCS.[5–9] Treatment-related risk factors for CEs include anthracyclines and radiation therapy to fields including the heart.[5, 6, 10–13] Other suggested risk factors are gender, age at cancer diagnosis,[5, 14] smoking habits, hypertension, diabetes mellitus[15] and genetic factors.[16, 17] The evidence concerning these non-treatment related risk factors for CEs is sparse and sometimes conflicting.

Previous studies on the evaluation of risk factors for different types of CEs are limited by small study samples, self-reported outcomes or outcomes based on record linkage without validation. Furthermore, detailed treatment information and information on contributing risk factors, such as lifestyle, is only investigated in a few studies.[15]

Knowledge of the incidence and risk factors for specific CEs is essential, as it can contribute to optimal follow-up care for CCS and it can inform recommendations for less toxic treatments in future childhood cancer patients. PanCareSurFup (PanCare Childhood and Adolescent Cancer Survivor care and Follow-up studies (PCSF); European Union (EU)-Grant agreement number 257505) is a collaboration of European cancer registries and clinical centres, that have agreed to pool their data.[18] PCSF, a 6-year study that started in 2011, aims to determine the incidence and risk factors of second cancers, late mortality and CEs, and to develop evidence-based clinical practice guidelines for long-term follow-up, transition to adult care and health promotion for CCS. The objective of this article is to describe the cohorts and the methodology of the cardiac study within the PCSF collaboration.

## Methods

### Cohorts of cardiac study

The cardiac study of the PCSF collaboration consists of a cohort study and a nested case-control study including eight European data providers (DPs): France, Hungary, Italy (two data providers), the Netherlands, Slovenia, Switzerland and the United Kingdom. The national committee protecting the confidentiality of the data (Commission Nationale de l’Informatique et des Libertés) approved this study. Tudományos kutatás etikai bizottság approved this study. Comitato Etico Interaziendale A.O.U. Città della Salute e della Scienza di Torino—A.O. Ordine Mauriziano—A.S.L. TO1 approved this study. Comitato Etico Regione Liguria III Sezione (pediatrica) approved this study. Institutional Review Board of the Academic Medical Center in Amsterdam approved this study National Medical Ethics Committee of the Republic of Slovenia approved this study. Ethics Committee of the Canton of Bern to the Swiss Childhood Cancer Registry approved this study. National Research Ethics Service UK approved this study. From the PCSF European cohorts we included 5-year CCS in whom cancer was diagnosed before 20 years of age.

### Methods of PCSF cardiac cohort study

#### Objective

The main objective of the cardiac cohort study is to determine the overall cumulative incidence and absolute risk for symptomatic CEs in European CCS. Furthermore, we will quantify the cumulative incidence of symptomatic CEs per childhood cancer type, different treatment modalities, age at treatment and calendar period of treatment. We also will assess the influence of childhood cancer type, different treatment modalities, age at treatment and calendar period of treatment on developing a symptomatic CE.

#### Primary outcomes

The CEs included in the cardiac cohort study are symptomatic heart failure, cardiac ischemia, pericarditis, valvular disease and arrhythmia graded according to the Criteria for Adverse Events (CTCAE)[19] as grade 3 (severe), 4 (life-threatening) or 5 (death) (Table 1). Any CE that does not meet these criteria is graded as ≤2. We use the extraction and flowchart method previously described to grade the CEs.[20] To avoid introducing a potential selection bias, we do not include asymptomatic CEs as these are mostly identified during follow-up care and not all CCS will undergo follow-up-care.

**Table 1:** Definitions of cardiac events (using CTCAEv3.0 and CTCAEv4.0)*.

	Grade 3	Grade 4	Grade 5
**Heart failure**	Symptomatic CHF responsive to intervention, or EF < 40%-20%, or SF <15%	Refractory CHF or poorly controlled; EF <20%; intervention such as ventricular assist device, ventricular reduction surgery, or heart transplant indicated; life threatening consequences CE*	Death due to heart failure
**Cardiac ischemia**	Symptomatic and testing consistent with ischemia or unstable angina or intervention needed	Myocardial infarction; life threatening consequences CE*	Death due to cardiac ischemia
**Pericarditis**	With physiological consequences CE (e.g pericardial constriction or pericardial effusion)	With life threatening consequences CE (e.g. hemodynamic compromise)	Death due to pericarditis
**Valvular disease**	Symptoms of severe regurgitation or stenosis, symptoms controlled with interventions	Life threatening consequences CE or intervention (e.g. valve replacement or valvuloplasty) indicated	Death due to valvular disease
**Arrhythmia**	Symptomatic and incompletely medically controlled or controlled with device (e.g. pacemaker, ICD or CRT)	Life threatening consequences CE (e.g. arrhythmia associated with CHF, hypotension, syncope, shock)	Death due to arrhythmia

*as reported in the Criteria for Adverse Events (CTCAE)v4.0

CHF = congestive heart failure; EF = ejection fraction; SF = shortening fraction; ICD = implantable cardioverse defibrillator; CRT = cardiac resynchronisation therapy

#### Data collection for baseline characteristics

The DPs collect the following data for all CCS included in the cohort analysis: gender, month and year of birth, month and year of first cancer diagnosis, morphology code, topography code, laterality and basis on which the first cancer diagnosis was made (histology, cytology, specific tumour markers or clinical investigation), and method of ascertainment of the first cancer diagnosis. In addition, the DPs collect information on surgery (yes/no), chemotherapy (yes/no), radiotherapy (yes/no), and/or a bone marrow transplant (yes/no), and the month and year of the start of treatment.

#### Statistical analyses

The outcome of interest is the occurrence of a symptomatic CE. We will consider the time at risk to start 5 years after first cancer diagnosis. To determine the absolute risk of the first occurring symptomatic CE and for the separate type of events, we will divide the sum of the number of events by the cumulative person years/ 10,000 in the total population (for the whole PCSF cohort as well as for the separate data providers). We will calculate the cumulative incidence of the first occurring symptomatic CE and of the separate types of CEs using competing risk analyses, with follow-up time since 5-year survival as the time scale. Competing risk analyses take into account that members die before developing a CE. If a cohort member died before developing a CE it was considered a competing risk. These CCS were not censored but left in the risk set with a certain follow-up duration, depending on the follow-up duration of CCS who did not develop a CE or did not die.[21] We will use Cox proportional hazards models, with attained age as time scale, to investigate the influence of gender, age at treatment, type of childhood cancer, type of treatment modality, and calendar period of treatment for the first occurring symptomatic CE and for the separate CE types.

### Methods of PCSF cardiac nested case-control study

#### Objective

The main objective of the nested case-control study is to determine the treatment-related risk factors for developing symptomatic CEs in CCS; both to confirm earlier identified risk factors and to identify new treatment, patient, lifestyle and co-morbidity related risk factors for CEs.

#### Study population

Each CCS identified in the cohort study with a validated CE is considered a “case”. “Controls” are randomly selected from survivors in the cohort study who did not develop a CE and these are matched to cases (ratio 1:1) on DP, gender, age at first cancer diagnosis, calendar year of first cancer diagnosis and length of follow-up after first cancer diagnosis. This procedure for sampling risk sets (i.e. density sampling) required controls to still be at risk at the time when the case developed the event. Thus, the length of follow-up (starting at first cancer diagnosis) in the control is at least that of the corresponding case. STATA (version 13, StataCorp) is used for control selection.

#### Detailed treatment data collection from medical records

In addition to the baseline data collected in the cohort study, detailed treatment data from all cases and controls is being collected. Data is collected from the medical records using an extraction form designed especially for PCSF (see also S1 Appendix). We collect data on the type of chemotherapy, cumulative dose (in mg/m^2^ or equivalent) and method of administration for each chemotherapeutic agent. For anthracyclines/ anthraquinones (doxorubicin, daunorubicin, epirubicin, idarubicin and mitoxantrone) we also collect data on infusion duration, dose per week and whether a cardio-protectant (like dexrazoxane) was given concurrently. We perform dosimetry for the whole body including seven points in the heart for all cardiac cases and controls who received radiotherapy, as previously described.[22] Furthermore, multiple imputation methodology was used when information was missing.

#### Detailed data collection from medical record and questionnaire

We collect data on potential confounding factors including congenital heart disorders (e.g. atrial/ ventricular septum defect, bicuspid aortic valve), hypercholesterolemia treated with medication, hypertension treated with medication, diabetes mellitus treated with medication or diet, clotting disease (protein S or C deficiency), thyroid disease treated with medication, pregnancies, lung transplant, kidney transplant, height, weight, waist circumference, hip circumference, family history of cardiac disease, physical activity, type of occupation, smoking and medication use. We collect these possible confounding factors by using questionnaires completed by patients or their families and subsequently DPs enter the data in a secure online database.

#### Collection of biomaterial

DNA data is being collected from the cardiac cases and cardiac controls that were alive and it is stored in the country of the DPs for future use. Blood samples are requested from CCS who visit an outpatient clinic and saliva/oral epithelial cells are requested by mail for those CCS who are not visiting an outpatient clinic, or for those CCS who received an allogeneic stem cell transplant.

#### Statistical analyses

For the separate types of CEs we will analyse the following covariates in our models: gender, age at primary childhood cancer diagnosis and different aspects of childhood cancer treatment (chemotherapy and radiotherapy). Since not all CEs have the same risk factors, each model will have different covariates based on the literature and clinical knowledge of each CE. We will investigate the role of anthracyclines [5, 6, 10, 11, 23], mitoxantrone [24, 25], cisplatin[26] as well as alkylating agents (as cyclophosphamide equivalence dose (CED)[27] and separate types of alkylating agents such as cyclophosphamide[28, 29]) as risk factors for CEs. We will also investigate the role of radiation therapy where the heart was part of the field[5, 6, 12, 13] and the role of cranial radiation therapy.[30, 31] The covariates and confounding factors will be considered in a conditional multivariate linear logistic model to control for pairwise matching in case-control studies.[32]

## Results

### Cohorts of cardiac study

In Table 2 the different cohorts are described. The PCSF cohort consisted of 59,915 5-year CCS with malignancies diagnosed between 1940 and 2009. Diagnoses were classified according to the International Classification of Childhood Cancer 3.[33]

**Table 2:** Inclusion criteria for each data provider within PCSF.

	Type of cohort (>5 year survivors)	Number of childhood cancer survivors in cohort	Age at primary cancer diagnosis	Type of malignancy	Period of primary cancer diagnosis
**France**	Hospital data (5 paediatric oncology centres), clinical trials, and cancer registry (ongoing)	3,097	0-<21 years	Solid tumours	1940–1986
**Hungary**	Hospital data, clinical trials, and nationwide cancer registry (ongoing)	5,162	0-<18 years	All, including benign CNS tumours	1971–2008
**Italy (hospital based)**	Nationwide cancer registry	3,004	0-<15 years	All	1960–2008
**Italy (population based) **	CCRP (Childhood Cancer Registry of Piedmont) and AIRTUM (Italian Association of Cancer Registries)	15,124	0-<18 years	All	1967–2009
**The Netherlands**	DCOG LATER (= Dutch Childhood Oncology group Long-term effects) registry based on Nationwide hospital based cohorts	6,087	0-<18 years	All	1964–2001
**Slovenia**	Nationwide Slovenian cancer registry, follow-up clinic	2,341	0-<16 years	All	1961–2002
**Switzerland**	Nationwide Swiss Childhood Cancer Registry	4,718	0-<21 years	All, and LCH	1964–2005
**United Kingdom**	Nationwide cancer registration	17,981	0-<15 years	All	1940–1991

CNS = central nervous system; LCH = Langerhans Cell Histiocytosis; PCSF = PanCareSurFup

### Primary outcome

The ascertainment of the CEs is described in Table 3. Different strategies were used to identify CEs: linkage to population/hospital or regional based databases (hospitalizations, medication use, general practitioner (GP) visits) as well as patient and GP based questionnaires. To obtain more information for the validation of the CEs, five DPs retrieved information from the medical records and the GP, and two DPs used the medical records and one used (telephone) questionnaires. Five of the eight DPs used the flowchart extraction method (20) to validate and grade the CEs. Cohort and nested case-control studies will be published separately.

**Table 3:** Cardiac outcome ascertainment per data provider within PCSF.

Data provider	Method of identification of potential cardiac events	Source of additional information to validate cardiac events
**France**	Questionnaires to patients/ medical records	Questionnaire/Telephone
**Hungary**	Visit to follow up clinic/	Medical records + GP
	questionnaires to patients	
**Italy (hospital based)**	Linkage: hospitalization database	Medical records
**Italy (population based)**	Hospital discharge database, medical records, questionnaire to patients	Medical records
**The Netherlands**	Questionnaires to patients or GP/Visit to follow up clinic/ medical records	Medical records + GP
**Slovenia**	Visit to follow up clinic/ questionnaires to patients	Medical records + GP
**Switzerland**	Visit to follow up clinic/ questionnaires to patients/ Linkage with death registry	Medical records + GP
**United Kingdom**	Questionnaires to patients/ linkage: different hospital episode databases (outpatient + in patient + emergency care + death registry)	Medical records + GP

GP = general practitioner; PCSF = PanCareSurFup

## Discussion

PCSF is an ongoing EU-funded collaborative research project investigating late effects in 5-year CCS. The cardiac study of PCSF examines cardiac late effects in a large cohort and in a nested case-control study with data from eight European DPs. The current manuscript describes in detail the methodology of this cardiac study and we highlight the unique features of this large Pan-European partnership. We believe that the cohort size and the chosen methodology of the cardiac study of PCSF will provide new evidence concerning risk factors of CEs in CCS.

The PCSF collaboration will add new knowledge to that gained in previously published studies.[5, 6, 8, 23, 34–37] In the past, CEs have been described in 3 types of studies. First, single-centre studies have assessed the incidence and risk factors of CEs in CCS. [6, 23, 36] Some of these studies have an almost complete follow-up and good outcome validation. However, due to their small sample sizes, these studies are not able to examine risk factors for all types of CEs.[6, 23, 36] Second, multicentre studies with large study populations have performed risk factor analysis per specific CEs, but these have mainly analysed self-reported outcomes that could be at risk of outcome reporting bias.[5, 35] Finally, nationwide studies using medical record linkage have a large study population with complete follow-up and a diminished risk of selection and outcome report bias [34, 38]. Nevertheless, they usually lack detailed treatment information. These missing data can prevent the possibility of performing an in-depth treatment related risk factor analysis.[34, 38] In contrast, PCSF addresses the incidence and risk factors of symptomatic CEs in a design that carries minimal risk of bias due to the method of ascertainment and extensive validation of CEs, as well as benefiting from detailed information on treatment.

Essential to the development of this project is the close collaboration between investigators of several European countries that provides systematically ascertained and validated data on symptomatic CEs. A potential drawback is that the differences in inclusion criteria and method of identification of potential CEs might mean that the data is too heterogeneous to pool. One of the main objectives of the cardiac component of PCSF is to adequately identify all potential risk factors for CEs. This is especially important for less frequent CEs for which we currently have only a small amount of data on risk factors. To adequately identify potential risk factors we need sufficient numbers for the separate types of CEs. Studies have shown that the number of outcome events are accountable for the number of covariates (potential risk factors) in the Cox proportional hazard model.[39] Less than 10 events per covariate in the results of the Cox proportional hazard should be interpreted with caution. We estimate that we will have 750 cardiac cases in the case-control study. Applying the classification of CE types described by a single centre clinical cohort study [6], we can estimate the number of the different types of CEs that we will have in our case-control study (Table 4). Hence, a strength of our study is that the final number of the different types of CEs will be sufficient for adequate risk factor analyses and safe interpretation.

**Table 4:** Estimated numbers of the separate types of CEs based on the assumption of 750 cardiac cases.

	Estimated number of each type of cardiac adverse event
Heart failure*	405
Cardiac Ischemia*	90
Pericarditis*	30
Valvular disease*	90
Arrhythmia*	135

*estimation based on van der Pal 2012: 54% heart failure, 12% cardiac ischemia, 4% pericarditis, 12% valvular disease and 18% arrhythmia.

CE = cardiac event

Previous studies have suggested a possible association between genetic factors and CEs.[16, 17] Thus, the DNA that PCSF is collecting from all the cardiac cases and cardiac controls in combination with the detailed information on the CCS will be a valuable source of information for future research. Furthermore, PCSF has focused on developing evidence-based long-term follow-up guidelines in collaboration with the International Guideline Harmonization group.[40] These guidelines will be used to guide survivors and health care providers on, amongst others, the prevention, early detection and treatment of long-term effects of childhood cancer. The recently published clinical practice guideline on cardiomyopathy surveillance for CCS notes the existing gaps in knowledge to improve cardiovascular health of CCS.[41] Armenian and colleagues highlighted the need for multidisciplinary and international collaboration with access to large populations in order to fill in these current research gaps.[41] Therefore, the findings of the cardiac cohort and nested case-control study of PCSF will be an important source of evidence and will provide an information base for long-term cardiac follow-up guidelines of CCS. Moreover, the successful identification of risk factors associated with the development of CEs can inform the further development of less cardiotoxic treatment for childhood cancer patients.

In conclusion, the cardiac studies included within the PCSF project benefit from the largest cohort of 5-year CCS with detailed treatment information and systematic ascertainment and validation of CEs. In order to achieve tailored follow-up of CCS at risk of CEs, the large number of individuals in PCSF will allow to adequately identify risk factors for different types of symptomatic CEs and incorporate this knowledge into evidence-based clinical practice guidelines for long-term follow-up of childhood cancer patients.

## Supporting Information

S1 Appendix Data collection form chemotherapy. (DOC)
