# Supplementary material for: Late Cardiac Events after Childhood Cancer: Methodological Aspects of the Pan-European Study PanCareSurFup
Source: PLoS One. 2016 Sep 19;11(9):e0162778. doi: 10.1371/journal.pone.0162778 (PMC5028033; doi:10.1371/journal.pone.0162778)
Supplement: Supplementary file 1 [file pone.0162778.s001.doc]

CACO-ID ___ - ___ - ___

PCSF-ID ___

Drug Name ___

Exit Date ___ - ___ - ___

FPN ___

Week No	Date Start (d d m m y y y y)	Date end (d d m m y y y y)	Dose in mg	Dose in mg/m²	Dose est?	Route	Hours
					Yes/No		
					Yes/No		
					Yes/No		
					Yes/No		
					Yes/No		
					Yes/No		
					Yes/No		
					Yes/No		
					Yes/No		
					Yes/No		
					Yes/No		
					Yes/No		
					Yes/No		
					Yes/No		
					Yes/No		
					Yes/No		
					Yes/No		
					Yes/No		
					Yes/No		
					Yes/No		
					Yes/No		
		Total dose:					
